# Application of non‐invasive bioreactance to assess hemodynamic function in patients with hypertrophic cardiomyopathy

**DOI:** 10.14814/phy2.15729

**Published:** 2023-06-18

**Authors:** Nduka Charles Okwose, Amy S. Fuller, Alaa I. Alyahya, Sarah J. Charman, Christopher Eggett, Peter Luke, Guy A. MacGowan, Djordje G. Jakovljevic

**Affiliations:** ^1^ Translational and Clinical Research Institute, Faculty of Medical Sciences Newcastle University Newcastle upon Tyne UK; ^2^ Research Centre for Health and Life Sciences, Institute for Health and Wellbeing, Faculty of Health and Life Sciences Coventry University Coventry UK; ^3^ Newcastle upon Tyne Hospitals NHS Foundation Trust Newcastle University Newcastle upon Tyne UK

## Abstract

Non‐invasive technologies have become popular for the clinical evaluation of cardiac function. The present study evaluated hemodynamic response to cardiopulmonary exercise stress testing using bioreactance technology in patients with hypertrophic cardiomyopathy. The study included 29 patients with HCM (age 55 ± 15 years; 28% female) and 12 age (55 ± 14 years), and gender matched (25% female) healthy controls. All participants underwent maximal graded cardiopulmonary exercise stress testing with simultaneous non‐invasive hemodynamic bioreactance and gas exchange. At rest, patients with HCM demonstrated significantly lower cardiac output (4.1 ± 1.3 vs. 6.1 ± 1.2 L/min; *p* < 0.001), stroke volume (61.5 ± 20.8 vs. 89.5 ± 19.8 mL/beat; *p* < 0.001), and cardiac power output (0.97 ± 0.3 vs. 1.4 ± 0.3watt; *p* < 0.001), compared to controls. At peak exercise, the following hemodynamic and metabolic variables were lower in HCM patients that is, heart rate (118 ± 29 vs. 156 ± 20 beats/min; *p* < 0.001), cardiac output (15.5 ± 5.8 vs. 20.5 ± 4.7 L/min; *p* = 0.017), cardiac power output (4.3 ± 1.6 vs. 5.9 ± 1.8 watts; *p* = 0.017), mean arterial blood pressure (126 ± 11 vs. 134 ± 10 mmHg; *p* = 0.039), and oxygen consumption (18.3 ± 6.0 vs. 30.5 ± 8.3 mL/kg/min; *p* < 0.001), respectively. Peak arteriovenous oxygen difference and stroke volume were not significantly different between HCM patients and healthy controls (11.2 ± 6.4 vs. 11.9 ± 3.1 mL/100 mL, *p* = 0.37 and 131 ± 50.6 vs. 132 ± 41.9 mL/beat, *p* = 0.76). There was a moderate positive relationship between peak oxygen consumption and peak heart rate (*r* = 0.67, *p* < 0.001), and arteriovenous oxygen difference (*r* = 0.59, *p* = 0.001). Functional capacity is significantly reduced in patients with HCM primarily due to diminished central (cardiac) rather than peripheral factors. Application of non‐invasive hemodynamic assessment may improve understanding of the pathophysiology and explain mechanisms of exercise intolerance in hypertrophic cardiomyopathy.

## 1 INTRODUCTION

Hypertrophic cardiomyopathy (HCM) is the most common genetic heart disease with great diversity in phenotypic expression (Maron et al., 2014). Exercise intolerance is a clinical hallmark in individuals with HCM (Hwang et al., 2022). Patients with HCM are less active compared to the general population with the choice to remain inactive often deliberate (Reineck et al., 2013). Multiple mechanisms may underpin exercise intolerance in these patients including chronotropic incompetence (Efthimiadis et al., 2011), under‐perfusion of peripheral musculature, diastolic dysfunction, mitral regurgitation, dynamic obstruction of the left ventricular outflow tract (LVOT) and systolic dysfunction in severe cases (Finocchiaro et al., 2015; Magrì & Santolamazza, 2017).

Cardiopulmonary exercise testing (CPET) has been employed for the evaluation of exercise capacity in patients with heart disease ensuring reliable estimation of oxygen consumption (VO_2_) and ventilator efficiency (O'Connor et al., 2009; Pandey et al., 2015; Saberi et al., 2017). However, limited number of studies have coupled non‐invasive techniques for evaluating cardiac function with CPET (Finocchiaro et al., 2015; Hwang et al., 2022) to adequately understand the determinants and pathophysiology of exercise intolerance in HCM. One of such non‐invasive techniques is bioreactance, which evaluates central hemodynamics based on an analysis of beat‐by‐beat changes (phase shifts) of an electric current moving through the thoracic cavity (Keren et al., 2007). Phase shifts occur due to pulsatile blood flow mainly from the aorta. Fluctuation in thoracic fluid volume produce variations in electrical capacitance and inductance referred to as bioreactance (Lee et al., 2011). Previous studies report bioreactance as superior for determining hemodynamic changes compared to thermodilution (Raval et al., 2008; Rich et al., 2013; Squara et al., 2007) with both sensitivity and specificity reported to be 93% (Squara et al., 2007).

The aim of this study was to determine exercise capacity in patients with HCM via CPET and to evaluate the hemodynamic determinants of exercise capacity in HCM patients, using bioreactance. We hypothesized that lower peak exercise cardiac pumping capacity indicated by cardiac output and cardiac power output may play a significant role in limiting exercise performance in this population.

## 2 METHODS

### 2.1 Study design

The present study which is a sub‐study of the SILICOFCM study (clinicaltrails.gov: NCT03832660) was a prospective, single‐center observational study of exercise tolerance in patients with hypertrophic cardiomyopathy compared to age and gender matched control participants. The study protocol was approved by the UK National Health Service, Northeast‐Tyne and Wear Research Ethics Committee (18/NE/0318) and the healthy control study was approved by Newcastle University Ethics Committee (2901/2017). All participants provided written informed consent before enrolment and all procedures were carried out according to the principles outlined in the Declaration of Helsinki and. Recruitment took place between June 2018 and February 2021.

### 2.2 Study participants

Participants were identified via the cardiology clinics of the Newcastle upon Tyne Hospitals NHS Foundation Trust. Diagnosis of HCM and inclusion criteria was based on the presence of significant left ventricular (LV) hypertrophy (end‐diastolic wall thickness > 15 mm at M‐mode or 2D echocardiography) in the absence of other etiologies, according to international criteria, or wall thickness between 13 and 15 mm, in the presence of abnormal electrocardiography, positive genetic test result or familial history of inherited cardiomyopathies (Ommen et al., 2020). Patients with LV systolic dysfunction were included in the study if there was a clearly documented history of HCM and preserved left ventricular ejection fraction (LVEF) in previous echocardiographic examinations.

Clinical genetic testing was performed at the genetic testing service and included the sequencing of at least eight sarcomeric genes including myosin binding protein C (MYBPC3), β‐myosin heavy chain (MYH7), essential and regulatory myosin light chains (MYL2, MYL3), cardiac troponin T (TNNT2), cardiac troponin I (TNNI3), α‐tropomyosin (TPM1), and cardiac actin (ACTC). Testing also included genes associated with metabolic cardiomyopathies to exclude Fabry's or Danon's disease. Major exclusion criteria included less than 3 months after septal reduction therapy, pacemaker, or ICD placement; clinical decompensation defined by NYHA class IV congestive heart failure symptoms; previously reported hypotensive response to exercise testing (≥20 mmHg decrease of systolic blood pressure from baseline blood pressure or an initial increase in systolic blood pressure followed by a decrease of systolic blood pressure ≥ 20 mmHg); resting blood pressure > 160/100 mmHg; use of. angiotensin converting inhibitors or angiotensin receptor blockers; resting LVOT gradient >50 mmHg; LVEF <55% quantified by echocardiography; renal insufficiency that is, glomerular filtration rate of less than 30 mL/min/1.73m^2^. Further exclusion for both groups were present or planned pregnancy; life expectancy <12 months; severe obesity that is, BMI >40 kg/m^2^; a history of exercise induced syncope or uncreated symptomatic ventricular arrhythmias; inability to exercise due to orthopedic or other non‐cardiovascular limitations and participation in competitive/ organized sport activities (e.g., football, basketball, rugby, hockey, etc.), burst activity (such as sprinting, racket sports, etc.) or heavy isometric exercise (such as body building or bench‐pressing) or opposition of refraining from the same for the duration of the study.

### 2.3 Echocardiographic and Doppler study

Echocardiographic images were acquired using Vivid IQ system (GE Healthcare UK). Real‐time images were acquired in the standard parasternal (long‐axis) and apical (apical four, apical two, and apical long) views, for which three cardiac cycles were recorded. Parasternal short‐axis views were acquired at three levels: basal (at mitral valve level), mid‐papillary, and apical (minimum cavity distal to papillary muscle level). LV diameter and fractional shortening, interventricular septum, and LV posterior wall thickness, and left atrial dimensions were measured using M‐mode and 2D echocardiography according to current recommendations (Lang et al., 2015). Peak velocity of the LVOT was recorded from the apical five chamber view by pulse Doppler, and pressure gradient was calculated. The sites and maximal extent of ventricular hypertrophy were assessed and measured in end‐diastole. LV volumes and LVEF and right ventricular systolic function were assessed from the apical 4‐chamber view, using the biplane method of discs. Left atrial volumes were measured in systole just before the mitral valve opening, using a monoplane area‐length method. Transmitral Doppler imaging was used to assess diastolic function.

### 2.4 Cardiopulmonary exercise testing procedure

Participants completed a maximal graded cardiopulmonary exercise test using a semi‐recumbent, electromagnetically controlled cycle ergometer (Corival, Lode & Groningen) with non‐invasive gas exchange (Cortex metalyser 3B). Exercise commenced with 3 min of unloaded cycling followed increased workloads of 10 watts per minute until maximum exertion was achieved. Bike cadence was maintained at 60–70 revolutions per minute throughout the test. Simultaneous 12‐lead electrocardiography (Custo, CustoMed) and an automated blood pressure (Tango, SunTech Medical) were recorded. Oxygen uptake (VO_2_), minute ventilation (VE), carbon dioxide production (VE/Vco2), and other CPET variables were acquired breath by breath, and expressed in 10‐s intervals. Exercise test was terminated (i) upon volitional exhaustion that is, inability to maintain a cadence of 60 rpm, or (ii) when maximum oxygen consumption was achieved, defined as the inability to increase oxygen consumption despite an increase in exercise intensity (watts) or (iii) terminated due to symptoms warranting termination of exercise.

### 2.5 Non‐invasive hemodynamic measurements

A bioreactance cardiography device (Starling SV, Baxter Healthcare) was used to determine cardiac output, stroke volume, and other hemodynamic variables at rest and during exercise. Information relating to methods and validation of the bioreactance technique have been reported previously (Fagnoul et al., 2012; Okwose et al., 2018; Raval et al., 2008; Rich et al., 2013). The system comprises a radiofrequency generator that creates a high‐frequency current that transmits across the thorax, four dual surface electrodes used to establish electrical body contact, an amplifier to record transthoracic voltage in response to the injected current, and circuitry for determining the relative phase‐shift between the injected current and the recorded voltage. Phase shifts arise from pulsatile blood flow primarily from the aorta. Volume changes in the thoracic cavity produce variations in electrical capacitance and inductance referred to as bioreactance (Lee et al., 2011). Estimation of cardiac output was based on the formula

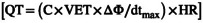

where C is a constant of proportionality, and VET is ventricular ejection time, which is determined from the bioreactance and electrocardiogram signals, ΔΦ/dt_max_ is the relative phase shift of current, and HR is heart rate. Hemodynamic variables were determined continuously at rest and during exercise and averaged over 10 s.

### 2.6 Statistical analysis

Results are expressed as mean ± SD for continuous variables or as percent for categorical variables. Comparison of groups was performed using two‐tailed unpaired *t‐*test. Pearson's coefficient of correlation was used to assess the relationship between oxygen consumption and hemodynamic function at rest and peak exercise. Statistical analysis was carried out using SPSS version 27.0 (SPSS Inc.).

## 3 RESULTS

**FIGURE 1:**
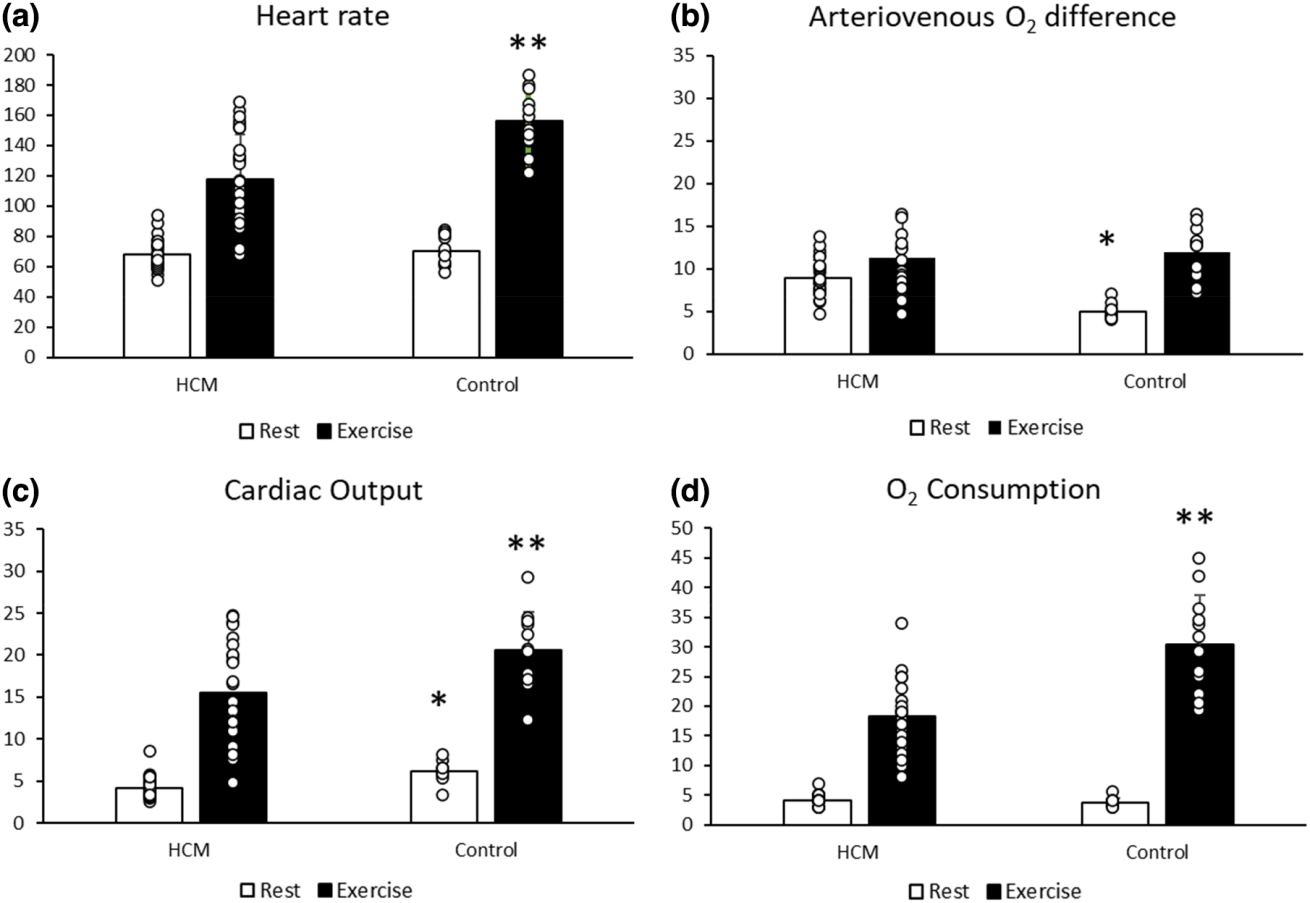
Hemodynamic and functional capacity variables measured at rest and peak exercise that is, Heart rate (b/min) (A) Arteriovenous oxygen difference (mL/100 mL) (B) Cardiac output (L/min) (C), Oxygen consumption (mL/kg/min) (D) between HCM patients (*n* = 29) and control (*n* = 12). Two‐tailed unpaired *t*‐tests; *p < 0.05 at rest and ** *p* < 0.05 at peak exercise.

One hundred and eighteen patients with HCM were screened by a cardiologist and 29 patients were successfully recruited. Twelve age‐ and sex‐matched controls were also recruited. Participants' demographic and clinical characteristics are presented in Table 1, while echocardiography measurements of patients with HCM are presented in Table 2. At rest, participants with HCM demonstrated lower cardiac output (4.1 ± 1.3 vs. 6.1 ± 1.2 L/min; *p* < 0.001), stroke volume (61.5 ± 20.8 vs. 89.5 ± 19.8 mL/beat; *p* < 0.001), cardiac power output (0.97 ± 0.3 vs. 1.4 ± 0.3 watt; *p* < 0.001) and mean arterial pressure (106 ± 10 vs. 103 ± 8 mmHg; *p* = 0.32), but higher arteriovenous oxygen difference (8.9 ± 2.7 vs. 4.8 ± 0.9 mLO_2_/100 mL; *p* < 0.001) compared to controls (Table 3). There were no differences between HCM patients and controls in resting heart rate (68 ± 10 vs. 71 ± 10 beats/min; *p* = 0.45), and oxygen consumption (4.2 ± 0.8 vs. 3.8 ± 0.7 mL/kg/min; *p* = 0.15), respectively. At peak exercise, the following hemodynamic and metabolic variables were lower in HCM patients compared to control that is, heart rate (118 ± 29 vs. 156 ± 20 beats/min; *p* < 0.001), cardiac output (15.5 ± 5.8 vs. 19.6 ± 5.5 L/min; *p* = 0.02), cardiac power output (4.3 ± 1.6 vs. 5.9 ± 1.8 watts; *p* = 0.005) and oxygen consumption (18.3 ± 6.0 vs. 30.5 ± 8.3 mL/kg/min; *p* < 0.001) (Table 3; Figure 1). Peak arteriovenous oxygen difference (11.2 ± 6.4 vs. 11.9 ± 3.0 mLO_2_/100 mL; *p* = 0.37) and stroke volume (131 ± 50.6 vs. 132 ± 37.7 mL/beat; *p* = 0.46) did not differ between HCM patients and healthy controls. There was a moderate positive relationship between peak oxygen consumption and peak heart rate (*r* = 0.67, *p* < 0.001), and arteriovenous oxygen difference (*r* = 0.59, *p* = 0.001) but not cardiac output and stroke volume, in HCM patients (Table 4).

**TABLE 1:** Participant demographic and clinical characteristics.

*Demographics*	HCM *n* = 29	Control *n* = 12	*p*
Age (years)	55 ± 15	55 ± 14	0.68
Female (%)	28	25	
Weight (Kg)	86 ± 18	79 ± 14	0.07
Height (cm)	173 ± 8	168 ± 8	0.14
Body mass index (kg m^−2^)	28.7 ± 5.1	28.0 ± 3.7	0.15
Medications, *n* (%)			
Beta blockers	20 (63)		
Diuretics	8 (25)		
Angiotensin converting enzyme inhibitor	7 (22)		
Angiotensin receptor blocker	3 (9)		
Statins	4 (13)		
Antiarrhythmics	10 (31)		
Anticoagulants	8 (25)		

*Note*: Data is expressed as mean ± SD. *p*‐Value; two‐tailed unpaired *t*‐test.

**TABLE 2:** Echocardiography characteristics of individuals with HCM (*n* = 29).

Variable	Mean ± SD
Left atrial diameter (mm)	41.0 ± 6.4
Left atrial volume (mL)	76.3 ± 45.0
Interventricular septum diameter (mm)	17.0 ± 4.5
Posterior wall diameter (mm)	10.0 ± 1.7
Left ventricular internal diameter in diastole (mm)	45.0 ± 6.1
Left ventricular internal diameter in systole (mm)	28.0 ± 6.2
Left ventricular ejection fraction (%)	63 ± 10
Early diastolic velocity ratio	10.1 ± 5.6
Left ventricular gradient rest (mm Hg)	6 ± 4
Left ventricular gradient peak exercise (mm Hg)	11 ± 5
Tricuspid annular plane systolic excursion (mm)	23.0 ± 5.5
Right ventricle systolic pressure RVSP (mmHg)	24 ± 10
Tricuspid annulus systolic velocity (m/s)	1.13 ± 0.27

**TABLE 3:** Rest and peak hemodynamic and metabolic measurements (HCM vs Control; *n* = 29/12),

*Parameter*	HCM	Controls	*p*‐Value
Rest			
Heart rate (b/min)	68 ± 10	71 ± 10	0.45
Stroke volume (mL)	61.5 ± 20.8	89.5 ± 19.8	<0.001
Cardiac output (L/min)	4.1 ± 1.3	6.1 ± 1.2	<0.001
Mean arterial blood pressure (mmHg)	106 ± 10	103 ± 8	0.32
Cardiac power output (Watt)	0.97 ± 0.29	1.4 ± 0.3	<0.001
Oxygen Consumption (mL/kg/min) Arteriovenous oxygen difference (mlO2/100 mL)	4.2 ± 0.8 8.9 ± 2.4	3.8 ± 0.7 4.9 ± 0.9	0.15 <0.001
Peak Exercise			
Heart rate (b/min)	118 ± 29	156 ± 20	<0.001
Stroke volume (mL)	131 ± 50.6	132 ± 37.7	0.76
Cardiac output (L/min)	15.5 ± 5.8	20.5 ± 4.7	0.017
Mean arterial blood pressure (mmHg)	126 ± 11	134 ± 10	0.039
Cardiac power output (Watt)	4.3 ± 1.6	5.9 ± 1.8	0.017
Oxygen consumption (mL/kg/min)	18.3 ± 6.0	30.5 ± 8.3	<0.001
Arteriovenous oxygen difference (mlO_2_/100 mL)	11.2 ± 6.4	11.9 ± 3.0	0.37

*Note*: Data is expressed as mean ± SD. *p*‐Value; two‐tailed unpaired *t*‐tests.

**TABLE 4:** Relationship between rest oxygen consumption and rest hemodynamic parameters in healthy/HCM (*n* = 29/12).

VO_2_ (mL/kg/min)		HR		SV		QT		MAP		CPO		AVO_2_diff	
		*r*	*p*	*r*	*p*	*r*	*p*	*r*	*p*	*r*	*p*	*r*	*p*
Rest	Control	−0.22	0.48	0.29	0.36	0.06	0.86	0.08	0.81	0.11	0.75	0.73	0.007
	HCM	−0.13	0.53	0.28	0.16	0.48	0.012	−0.12	0.55	0.51	0.008	0.05	0.79
Peak exercise	Control	0.61	0.04	0.49	0.88	0.29	0.34	0.09	0.77	0.27	0.41	0.58	0.049
	HCM	0.67	<0.001	−0.05	0.82	0.16	0.44	0.04	0.86	0.36	0.09	0.59	0.001

Abbreviations: AVO_2_diff, arteriovenous oxygen difference; CPO, cardiac power output; HR, heart rate; MAP, mean arterial pressure; *p*, *p*‐Value; QT, cardiac output; *r*, Pearson's degree of correlation; SV, stroke volume; VO_2,_ oxygen consumption.

## 4 DISCUSSION

The present study aimed to determine hemodynamic factors responsible for exercise intolerance in individuals with HCM using continuous non‐invasive monitoring technology. Our results show that individuals with non‐obstructive HCM can compensate a deficit in resting stroke volume at maximal exercise, but this is inadequate to significantly improve exercise capacity. Cardiac output is reduced at peak exercise due to significant attenuation of heart rate with no difference in skeletal muscle oxygen extraction which suggests that cardiac, rather than peripheral factors are primarily responsible for exercise intolerance in this cohort of HCM patients. These results show the utility of exercise testing coupled with non‐invasive hemodynamic monitoring to determine exercise response in patients with HCM.

The monitoring and optimization of cardiac output are vital aspects of hemodynamic management in patients with varied heart conditions. Over the past two decades, concerted efforts have been made to develop non‐invasive technologies to analyze flow and response to fluid therapy without losing significant accuracy and precision, thereby avoiding the complications caused by invasive techniques (Scheeren & Ramsay, 2019). In the current study, bioreactance technology was used to continuously monitor hemodynamic function at rest and during exercise with readings averaged over 10 s indicating proficiency to detect rapid changes in cardiac output.

Peak oxygen consumption, an outcome of CPET, is an important marker in the management of HCM and prediction of prognosis (Finocchiaro et al., 2015). It is primarily influenced by central (cardiac) and peripheral factors (skeletal muscle function) (del Buono et al., 2019; van Wezenbeek et al., 2019). Reduced exercise capacity (i.e., peak exercise O_2_ consumption <22 mL/kg/min) reported in the present study is consistent with previous studies (Saberi et al., 2017; Wu et al., 2019). Reduced cardiac output rather than arterio‐venous oxygen difference was the main factor responsible for lower exercise tolerance in this study. Cardiac output was significantly reduced possibly due to attenuation of heart rate caused by beta blocker medication. It has been suggested that HCM patients treated with beta blockers have baroreflex sensitivity comparable to healthy individuals, thus effectively adapting to an alteration in vagal tone triggered by a preceding change in blood pressure (Katarzynska‐Szymanska et al., 2013). Whilst this assumption supports our findings under resting conditions, beta‐blockers have been reported to attenuate blood pressure response to exercise (Chen et al., 1999). Wu et al., 2019; however, reported reduced exercise capacity even after withdrawal of beta blocker medication. Earlier studies suggest that peak exercise cardiac output appears to be a major determinant of peak exercise capacity in patients with hypertrophic cardiomyopathy, which is dependent primarily on stroke volume augmentation (Lele et al., 1995; Re et al., 2013). In contrast, neither peak cardiac output nor peak stroke volume showed any relationship with peak oxygen consumption in the present study. Interestingly, data showed a moderate positive relationship between peak oxygen consumption and peak heart rate, and peak arteriovenous oxygen difference. In the early phase of left ventricular systolic dysfunction, activation of the sympathetic nervous system can lead to short‐term regulation of central and peripheral hemodynamics, which will ultimately become pathological (Kishi, 2012). Notwithstanding, our observation is unable to confirm if increase in stroke volume is compensatory and would lead to long‐term pathological consequences. The patients in this study presented with non‐obstructive HCM and preserved ejection fraction. This group is not well‐characterized as prior studies included patients with left ventricular outflow obstruction (Hwang et al., 2022; Saberi et al., 2017). Our study participants predominantly complained of New York Heart Association class II symptoms with no formal diagnosis of heart failure. Interestingly, commonly observed phenotypic markers of heart failure preserved ejection fraction, such as left atrial enlargement was borderline normal, and atrial fibrillation present in 18% of participants. The natural history of disease progression in this group of patients vary and is multifactorial. We speculate that it represents an important group in which interventions may be targeted with higher yield to prevent or delay the transition to heart failure, where changes in the properties of the ventricle and vasculature may be irreversible. Since catheterization may not be applicable in majority of HCM patients, based on our experience and results degenerated we would recommend the use of non‐invasive hemodynamic assessment at rest and in response to exercise to help improve understanding of physiology and pathophysiology in these patients.

### 4.1 Limitations

Firstly, our sample size was relatively small compared with the number of participants screened. This was majorly due to the COVID‐19 pandemic which resulted in widespread lockdowns and shutting down of our testing facility for several months. Secondly, bioreactance is an indirect measure of cardiac output and the addition of another gold standard comparative technique in this population would have verified our findings. However, adding an invasive technique such as thermodilution or the Fick's principle was impractical and ethically unjustifiable for our study population. Thirdly, our sample may not represent the general HCM population, as patients with more advanced disease were under‐represented. Notwithstanding, we have shown with our sample that significant functional limitations exist even at the early stages of HCM.

In conclusion, individuals with non‐obstructive HCM display reduced exercise capacity underlined by central (cardiac) rather than peripheral factors. Hemodynamic changes during exercise can be continuously monitored using non‐invasive bioreactance technology. Future research is required to examine whether clinical progression of disease can be delayed or prevented through interventions targeted to this group of patients.

## AUTHOR CONTRIBUTIONS

*Study concept and design*: Djordje G. Jakovljevic, Guy A. MacGowan, Nduka Charles. Okwose; *Study Supervision*: Djordje G. Jakovljevic, Christopher Eggett, Guy A. MacGowan; *Acquisition of data*: Amy S. Fuller, Alaa I. Alyahya, Sarah J. Charman, Peter Luke; *Analysis and interpretation of data*: Nduka Charles. Okwose, Sarah J. Charman, Djordje G. Jakovljevic; *Drafting of the manuscript*: Nduka Charles. Okwose, Sarah J. Charman, Djordje G. Jakovljevic: *Critical review of the manuscript*: all authors; Guarantors and take responsibility for the content of the manuscript, including the data and analysis: Djordje G. Jakovljevic, Nduka Charles. Okwose. All authors approved the final version of the manuscript for submission.

## FUNDING INFORMATION

This work has been conducted as part of the SILICOFCM project which received funding from the European Union's Horizon 2020 Research and Innovation Programme under Grant Agreement No. 777204. The study was also supported by the Investigator Research Initiated Grant provided by the Baxter Healthcare Corporation. DGJ and NCO are supported by the European Horizon 2020 Research and Innovation Programme under the grant agreement number 952603. The design of the study, data collection, analyses, interpretation of data, and drafting of the manuscript do not reflect the views and opinions of the funders.

## CONFLICT OF INTEREST STATEMENT

The authors declare that they have no conflicts of interests.

## DATA AVILABILITY STATEMENT

The corresponding author shall provide data upon reasonable request.
